# Effect of Dry Heat Application on Perineal Pain and Episiotomy Wound Healing among Primipara Women

**DOI:** 10.1155/2023/9572354

**Published:** 2023-01-04

**Authors:** Naglaa Zaki Hassan Roma, Rasha Mohamed Essa, Zohour Ibrahim Rashwan, Afaf Hassan Ahmed

**Affiliations:** ^1^Obstetric and Gynecological Nursing Department, Faculty of Nursing, Alexandria University, Alexandria, Egypt; ^2^Obstetric and Gynecological Nursing Department, Faculty of Nursing, Damanhour University, Damanhour, Egypt; ^3^Pediatric Nursing Department, Faculty of Nursing, Alexandria University, Alexandria, Egypt; ^4^Nursing Department, College of Health Sciences, University of Bahrain, Zallaq, Bahrain

## Abstract

**Background:**

Women who undergo perineal episiotomy can be affected by several complications such as bleeding, infection, perineal pain, dyspareunia, reduction of sexual desire, as well as urinary and anal incontinence. Perineal pain related to episiotomy has been reported to interfere with women's daily activities postpartum and can prevent proper breastfeeding, proper rooming-in, and maternal-infant bonding. The purpose of this study was to determine the effect of dry heat application on perineal pain and episiotomy wound Healing among primipara women.

**Method:**

A quasi-experimental, two-group, pre-post-test research study was conducted at the postnatal inpatient ward and the outpatient clinic of the El-Shatby Maternity University Hospital in Alexandria. A sample of 100 parturient women was divided into the following two groups at random: dry heat and moist (control) heat. Women in the moist heat group were advised to sit in a basin (tub) of warm water for 10 minutes, while those in the dry heat group were instructed to set an infrared light (230 volts) at a distance of 45 cm from the perineum after 12 hours post episiotomy. Both interventions were applied twice a day for ten consecutive days. They evaluated the severity of their perineal pain at baseline and repeated it on the 5^th^ and 10^th^ days after obtaining the interventions while the episiotomy wound healing was assessed on the 5^th^ and 10^th^ days.

**Results:**

It was discovered that the dry heat group had a significantly improved episiotomy wound healing as regards perineal redness, edema of the perineal area, ecchymosis, wound discharge, and approximation of wound edges on the 5th (*P* < 0.001, *P* < 0.001, *P* < 0.007, *P* < 0.003, and *P* < 0.001, respectively) and 10th day after intervention (*P* < 0.001, *P* < 0.001, *P* < 0.001, *P* < 0.005, and *P* < 0.001, respectively) than the moist heat group. The primipara women had significantly lower perineal pain intensity in the dry heat group on the 5th and 10th days after intervention than in the moist heat group (^MH^*P* < 0.001 for the dry heat group and ^MH^*P* = 0.004 for the moist heat group).

**Conclusion:**

The application of dry heat promoted episiotomy wound healing among primipara women and reduced their perineal pain during early postpartum days than moist heat.

## 1. Introduction

Although the postpartum period is a wonderful and joyful experience for mothers, it is life-challenging and full of many stressful events [1]. Many health problems may arise during the postpartum period and, if not treated promptly and effectively, can lead to ill health and even death of the mother, her neonate, or both [2]. Perineal discomfort is the most common problem encountered in the postnatal period. It occurs in 42% of women after spontaneous delivery and persists after the first three months. Perineal pain worsens following instrumental delivery, spontaneous tears, or episiotomy [3].

An episiotomy or perineotomy is a deliberate surgical incision to the perineum, made either during the second stage of labor or just before delivery, to widen the vaginal orifice [4]. It is the most common surgical procedure performed during the labor of primiparous women and was introduced to clinical practice in the eighteenth century [5]. There are four types of episiotomies; median, mediolateral left or right, lateral, and J-shape [6]. Globally, the prevalence of episiotomy has a wide geographic variation between countries and institutions because of differences in attitudes and training. Up to now, high episiotomy rates are still being reported in many countries [7]. In Egypt, according to the Statistical Department of the postnatal inpatient ward and the outpatient clinic of the El-Shatby Maternity University Hospital, the episiotomy rate was 94% of vaginal births in 2015, while in 2016 it was 93% of vaginal births [8]. Despite the World Health Organization [9] recommending a reduction in the episiotomy rate to 10% for normal vaginal deliveries, the procedure is still performed by 30% to 50% of women [9]. Episiotomy is associated with clinically relevant morbidities for newly delivered women including perineal trauma, the need for suturing, and healing complications [4].

Episiotomy-related morbidity can influence the physical, psychological, and social well-being of women, both in the immediate and long-term postnatal period [10]. Women who undergo perineal episiotomy can consequently be affected by several complications, such as bleeding, infection, damage to the anal sphincter and mucosa, wound opening, dyspareunia, reduction of sexual desire, perineal pain, as well as urinary and anal incontinence. Perineal pain and discomfort related to episiotomy have been reported to interfere with women's daily activities, such as sitting, walking, and lifting the neonate. Moreover, it can interrupt proper breastfeeding, rooming-in, as well as maternal-infant bonding [11].

Postpartum is a sensitive time when mothers must juggle their recovery while dealing with the needs of their newborns. Effective episiotomy management is a major aspect of postpartum care that can positively affect women's lives [12]. Several interventions are available for relieving perineal discomfort, promoting healing, and reducing perineal edema, redness, and pain. These interventions include perineal hygiene, keeping the wound dry, and using various pharmacological and nonpharmacological treatments [13]. The maternity nurse should be aware of efficient episiotomy wound management which can decrease the suffering of postpartum mothers and improve healing. Many nonpharmacological measures are assisting the healing process, which encompasses acupuncture, cryotherapy, laser therapy, electrical stimulation, the performance of Kegel's exercise, dry heat (infrared therapy), and moist heat topical applications (sitz bath) [14].

Moist heat is one of the techniques that can alleviate perineal pain and enhance wound healing. It requires immersion of the perineal area and buttocks for 15 to 20 minutes in warm water at a temperature of 110°–115°F. It can be used to lessen perineal discomfort, itching, or muscle spasms [15]. Moist heat can also facilitate wound healing by soaking the perineum and anus, improving circulation, decreasing edema, and inflammation, and promoting muscle relaxation [16]. It seems that heat stimulates heat receptors of the skin and deeper tissues, and it may reduce pain, as proposed in gate control theory [17]. On the other hand, dry heat using the infrared lamp is a special type of therapy in which episiotomy is treated using the healing effect of light. At a distance of 45–50 cm and for 10–15 minutes, the incision site or the diseased portion of the perineum is exposed to infrared radiation with a light source of 230 volts. Therefore, these rays relax muscles, promote circulation, and provide pain relief [18]. Recently, studies have shown conflicting findings on the most effective strategies to alleviate pain from episiotomy and improve wound healing [19, 20]. Therefore, this study was carried out to compare the effects of dry heat versus moist heat application in reducing perineal pain and promoting episiotomy wound healing among primiparous women.

### 1.1. Aim of the Study

This study aimed to determine the effect of dry heat application on perineal pain and episiotomy wound Healing among primipara women.

### 1.2. Research Hypothesis

Primipara women who receive dry heat application on their episiotomy wound exhibit episiotomy wound healing and lower perineal pain intensity than those who receive moist heat.

### 1.3. Research Design and Setting

A quasi-experimental, two-group, pre-post-test research study was conducted at the postnatal inpatient ward and the outpatient clinic of the El-Shatby Maternity University Hospital in Alexandria. This setting was chosen as it is the largest maternity health agency in Alexandria and also episiotomy procedure is routinely performed on all primipara attending for delivery.

### 1.4. Subjects

The study used a convenience sample of 100 parturient women who met the following inclusion criteria; had a normal vaginal delivery with episiotomy; primipara during the first 2 hours after delivery; complained of perineal discomfort (pain); and agreed to participate in the study. While illiterate women and those who used any pain-relieving drug (painkillers may mask the impact of the intervention), had labor or postpartum complications, diabetes and anemia were excluded from the study.

In the beginning, the researchers spoke with 900 parturient women to identify individuals who fit the prerequisites. 127 parturient women were among the instances that were found. The sample size was calculated using the Epi Info program version 10 with the following parameters: a population size of 127, a confidence level of 95%, an expected frequency of 50%, and an acceptable error of 5%. 100 parturient women made up the required minimum sample size.

**Figure 1:**
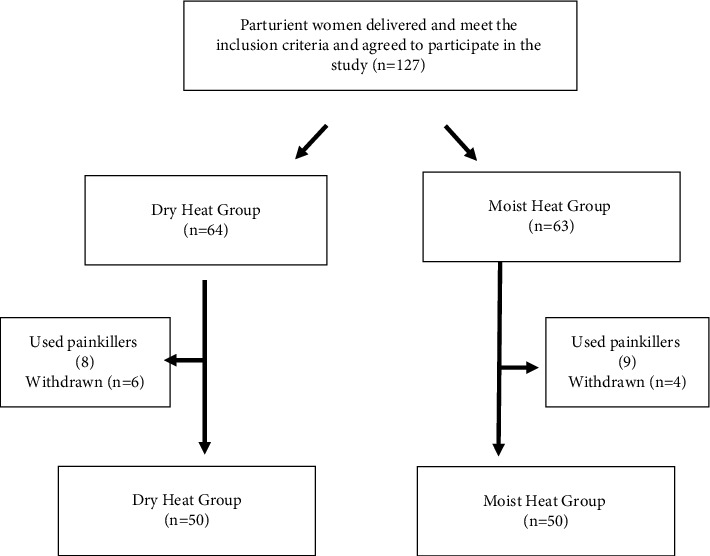
Flow chart of participants' recruitment process.

During the study period (January 2021–May 2021), a sample of 100 out of 127 parturient-eligible women was randomly assigned to two equal parallel groups in the postpartum inpatient ward and the outpatient clinic from 8 AM to 1 PM, three days a week. As shown in Figure 1, one participant was placed in the dry heat group, while the next was placed in the moist heat group.

### 1.5. Instrument

#### 1.5.1. Tool I: Visual Analog Scale (VAS)

- This tool was adopted by Melzack and Katz (1994) to measure pain intensity. It is a self-reported scale consisting of a horizontal line used for the subjective estimation of a patient's pain. It is comprised of a 10-point numerical scale, corresponding to the degree of pain, with zero representing no pain and 10 representing the worst degree of pain. Scores 1, 2, and 3 indicate mild pain, while scores 4, 5, and 6 indicate moderate pain, and scores 7, 8, and 9 indicate severe pain; finally, a score of 10 indicates the worst unbearable pain [21].
- The sociodemographic data such as age, level of education, occupation, current residence, and type of family as well as data about episiotomy such as indications of episiotomy and type of episiotomy were attached to this tool.

#### 1.5.2. Tool II: The Standardized REEDA Scale (REEDA)

- The REEDA scale was originally developed by (Davidson, 1974). Then, it was adapted by (Alvarenga et al., 2015). It is an observational checklist used for assessing episiotomy wound healing. It can be used to assess all types of postpartum perineal trauma. It has five components, namely, redness, edema, ecchymosis, discharge, and approximation of the wound edges. Each component takes a score ranging from 0 to 3 as follows:
- Redness: 0 = none, 1 = mild within 0.25 cm of incision, 2 = moderate within 0.5 cm of incision bilaterally, and 3 = severe beyond 0.5 cm of incision bilaterally.
- Edema: 0 = none, 1 = mild perineal, less than 1 cm from the incision, 2 = moderate perineal and\or vulvar, between 1 and 2 cm from the incision, and 3 = severe perineal and\or vulvar, greater than 2 cm from an incision
- Ecchymosis: 0 = none, 1 = mild within 0.25 cm bilaterally or 0.5 cm unilaterally, 2 = moderate between 0.25 and 1 cm bilaterally or between 0.5 and 2 cm unilaterally, and 3 = severe greater than 1 cm bilaterally or 2 cm unilaterally.
- Discharge: (0 = none, 1 = serous, 2 = serosanguinous, and 3 = bloody, purulent)
- Approximation: 0 = closed, 1 = mild skin separation of 3 mm or less, 2 = moderate skin and subcutaneous fat separation, and 3 = severe skin and subcutaneous fat and fascial layer separation).
- Total REEDA score ranges between 0 and 15. A higher score indicates poor wound healing while a lower score indicates good wound healing. The total score of the REEDA scale was categorized as follows:
- Completely healed from 0 to 2
- Moderately healed from 3 to 5
- Mildly healed from 6 to 8
- Not healed from 9 to 15

The kappa coefficient was used in the reliability analysis of the REEDA scale by Alvarenga et al. (2015), where the discharge item (0.75 < Kappa ≥ 0.88), assessment of edema (0.16 < Kappa ≥ 0.46), ecchymosis (0.25 < Kappa ≥ 0.42), and redness (0.46 < Kappa ≥ 0.66). For the item correspondence, the agreement decreased from excellent in the first assessment to good in the last assessment. In the fourth evaluation, the assessment of all items displayed excellent or good agreement among the evaluators.

## 2. Method

The parturient woman was initially addressed by the researchers, who established a report and collected the sociodemographic information during the initial 15–20 minute interview that occurred within the first two hours after delivery at the hospital stay in the postpartum inpatient ward. Additionally, the degree of episiotomy wound healing and baseline perineal pain intensity were evaluated. Following the evaluation, the researchers gave the participants a health education session while using illustrative pamphlets for both groups.

### 2.1. Interventions

For the moist heat group, women were encouraged to sit in a basin (tub) of warm water (110°–115°F) without pressure on the perineum and with their feet flat on the floor for 10 minutes twice a day for ten consecutive days [15]. The researchers demonstrated to each woman how to do a warm sitz bath, and it was followed by demonstrations and discussions. After 12 hours of episiotomy, this procedure was carried out in the morning and evening for ten consecutive days [15].

For the dry heat group, an infrared lamp was placed at a distance of 45 cm from the perineum, and the heat was produced at 230 volts for ten minutes. But the women were checked after the first five minutes to make sure that the heat temperature was suitable. The researchers demonstrated to each woman how to use the infrared lamp, and it was followed by redemonstrations and discussions. After 12 hours of episiotomy, this procedure was carried out in the morning and evening for ten consecutive days. The researcher gave the infrared lamp device to each woman and then restored it after the completion of the study.

The researchers provided each woman with health education regarding the value of follow-up at the conclusion of the session in order to ensure compliance with the interventions they had assigned to them and to evaluate wound healing. Through daily phone calls, the researchers encouraged the women to carry out the interventions they had given them by reassuring them of their advantages and the necessity of follow-up.

### 2.2. Follow-Up

The researchers contacted the parturient women of the two groups daily and ascertained that they had performed the interventions. They were also instructed to attend the outpatient clinic of the El-Shatby Maternity University Hospital on the 5^th^ and 10^th^ days after the first session during the morning shift for follow-up where the episiotomy wound healing process and perineal pain intensity were reassessed. The perineal area was observed for redness, edema, ecchymosis, discharge, and approximation of the skin as well as perineal pain.

### 2.3. Ethical Considerations

On 13 December 2020, approvals for performing the study were gotten from the Ethical Research Committee review board of the Faculty of Nursing, Alexandria University, and ClinicalTrials.gov also reported the study as having received approval (https://clinicaltrials.gov/ct2/show/NCT05186532). The relevant authorities of the studied area provided the researchers with authorization to perform the study. The 7th revision of the Declaration of Helsinki's Principles guided the study's conduct (World Medical Association, 2013). Prior to implementing the interventions, the researchers spoke with parturient women who met the inclusion requirements and gave them a thorough explanation of the nature of the interventions, their advantages, and any potential hazards. Additionally, researchers confirmed that participation in the study is completely voluntary. Also highlighted was their ability to decline participation or exit from the study at any moment without any impact on the quality of treatment they got. The anonymity and privacy of the obtained women were guaranteed. The participants completed a written informed consent form after reaching an agreement.

### 2.4. Statistical Analysis

Data analysis was done using SPSS version 20.0 (Statistical Package for Social Sciences). Descriptive statistics such as frequencies, percentages, mean, and standard deviations were used to describe parturient women's sociodemographics. Data were tested for normality using the Kolmogorov–Smirnov test, and all variables showed non-normal distribution. As for differential statistics, a comparison between the parturient women in the two studied groups regarding their mean age was made using the *T*-test (*T*), The severity of perineal pain before and on the fifth and tenth days after the interventions, as well as the evaluation of episiotomy wound healing on the fifth and tenth days, were all assessed using Mann–Whitney (*Z*) tests. All of the statistical analyses were considered significant at *P* < 0.05.

## 3. Results

Table 1 displays that 58% of parturient women in the dry heat group and 64% of the moist heat group were 20 to less than 30 years old. The mean age of parturient women in the dry heat group was 26.444 ± 4.785 years and 25.08 ± 5.014 years in the moist heat group. The vast majority (92% and 86%) of them, respectively, were housewives. However, majorities (82% and 72%) of dry and moist heat groups were rural residents, about two-fifths (46% and 40%) of them, respectively, live with their extended family.

**Figure 2:**
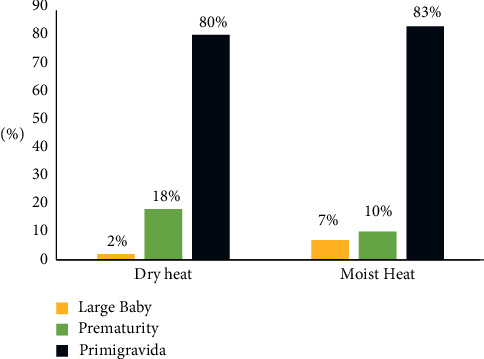
Graphical presentation of dry and moist heat groups according to their indications of episiotomy.

Figure 2 exhibits that in about three-quarters of dry heat and moist heat groups, respectively (80% and 83%), their indication of episiotomy was primipara.

**Figure 3:**
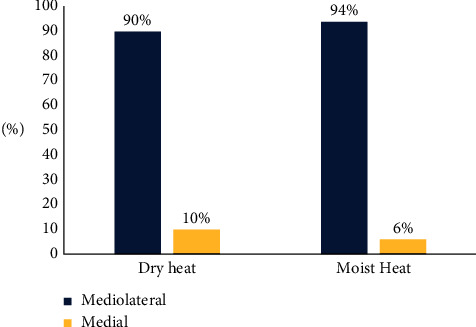
Graphical presentation of dry and moist heat groups according to their type of episiotomy.

It is evident from Figure 3 that most of the dry and moist heat groups, respectively (90% and 94%), had Mediolateral episiotomy.

Table 2 reveals that 28% and 40% of parturient women in the dry heat group and 38% and 36% in the moist heat groups, respectively, had experienced moderate and severe perineal pain intensity before applying the interventions. On the 5^th^ day after the intervention, there was an obvious decline in perineal pain intensity among both groups in favor of the dry heat group, where 24% of the dry heat group had severe perineal pain intensity, compared to 28% of the moist heat group, respectively. However, on the 10^th^ day after the intervention, it was observed that only 6% of the dry heat group had severe pain compared to 10% of the moist heat group. Significant differences were found between the within groups on the 5^th^ day and 10^th^ day (^MH^*P* < 0.001 for the dry heat group and ^MH^^*P*=0.004^ for the moist heat group).

Table 3 manifests there were no significant differences between both groups regarding all components of the REEDA scale before intervention (*P*=0.89, *P*=0.96, *P*=0.82, *P*=0.5, and *P*=0.5, respectively). On the 5^th^ day after intervention, there was a statistically significant difference between both groups on 5^th^ day after intervention as regards all components of the REEDA scale as presented by redness, edema, ecchymosis, discharge, and approximation where (*P* < 0.001, *P* < 0.001, *P* < 0.007, *P* < 0.003, and *P* < 0.001, respectively). Furthermore, significant differences were observed between both groups on the 10^th^ day after intervention as regards all components of the REEDA scale as presented by redness, edema, ecchymosis, discharge, and approximation where (*P* < 0.001, *P* < 0.001, *P* < 0.001, *P* < 0.005, and *P* < 0.001, respectively).

Table 4 shows an obvious decline in the mean perineal healing scores among the dry and moist heat groups on the 5^th^ day (2.34 ± 1.661 and 6.24 ± 3.274) and 10^th^ day after the interventions (0.84 ± 0.739 and 3.32 ± 2.645), with highly significant differences (*P* < 0.001) between them in the favor of the dry heat group. Where, on the 5^th^ day after intervention more than three-quarters (78%) of the dry heat group had complete healing of episiotomy wounds compared to only 20% of the moist heat group. Furthermore, on the 10^th^ day after intervention, most (96%) of the dry heat group did achieve complete healing of episiotomy wounds compared to 52% of the moist heat group. Significant differences were found within the dry heat group (*P* < 0.001^c^) and within the moist heat group (*P* < 0.021^a^) on the 5th day and the 10th day after the interventions, respectively, in favor of the dry heat group.

## 4. Discussion

Giving birth is a powerful and life-changing event with a lasting impact on women and their families. To facilitate the birthing process and prevent perineal tears during vaginal delivery, episiotomy is done. Unfortunately, such a kind of wound is associated with many complications. Women who underwent this procedure were at greater risk for greater blood loss during labor, delayed wound healing, and increased perineal pain during the early postpartum period. Moreover, poor episiotomy healing can influence the physical, psychological, and social well-being of women throughout the postnatal period [10]. With proper episiotomy care, the infection can be prevented and healing takes place more quickly. Healthcare is a dynamic field where maternity nurses are ever-spiraling towards greater improvement and adopting innovative technologies and interventions. Among these interventions, dry heat and sitz baths are the most effective methods of relieving episiotomy discomfort, and pain as well as fostering wound healing [22]. The secondary outcomes of such interventions were to improve women's quality of life, fasten their resume to daily life activities and decrease episiotomy-related morbidity. Moreover, alleviating perineal pain enables the mothers to sit comfortably and assume a proper breastfeeding posture that ultimately would enhance the mother-infant bond.

On investigating the perineal pain level, the results of the present study revealed that the pain was significantly reduced in the dry heat group than in the moist heat group on the 5^th^ and 10^th^ days after the interventions. The pain reduction could be attributed to the fact that heat application induces vasodilatation and increases blood circulation to the area. This could enhance tissue oxygenation, reduce muscle spasms, accelerate waste product removal, reduce inflammation, and promote episiotomy wound healing. Moreover, the heat application had soothing effects on the superficial sensory nerve endings. The results of the present study agree with the findings of Boddupalli [23]; who reported that the intensity of pain decreased with the infrared light fomentation on episiotomy, and pain relief was seen at the end of the 4^th^ day of follow-up. The author further added that dry heat is superior due to the fact that it continues for a longer duration than a moist one, keeps the wound dry, and improves healing. The present finding is also consistent with the study done by El-Lassy and Madian [24]; who found that the pain mean score was statistically significantly lower after the application of infrared lamp therapy among the study group than the control one. In addition, Gomathi et al. [25] reported that the infrared light application was effective in relieving pain levels among postnatal mothers. They added that Infrared rays have a therapeutic effect in aggregating the blood supply and releasing the pain. Moreover, a similar result was observed in Rani's [26] research, which found that there was a significant reduction in episiotomy pain score in the experimental group after infrared radiation therapy than in the control group. In addition, [27] concluded that both dry heat and moist heat interventions were effective, but dry heat was more effective than moist heat with sitz baths in reducing the severity of episiotomy-associated pain among postnatal mothers.

On the contrary, Chandraleka et al. [19] indicated that the sitz bath is more effective in reducing the level of episiotomy pain among postnatal mothers. They attributed the pain relief to the sedative effect of the warm water sitz bath that inhibits perineal irritation and itching in the genital area. It also prevents soreness and burning sensations around the perineum, which helps in reducing pain, itching, and discomfort. The present study's findings also contradict Huang et al. [28]; who investigated the effect of far-infrared radiation on perineal wound pain and sexual function in primiparous women undergoing an episiotomy. The study revealed no significant difference between the intervention and comparison groups regarding the perineal pain intensity immediately after delivery, one week, or six weeks postpartum.

Concerning episiotomy wound healing using the REEDA scale, the present study indicated highly significant statistical differences between both groups in favor of the dry heat group on the 5^th^ and 10^th^ days after the intervention. This improvement in episiotomy wound healing may be due to the penetration of the emitted infrared light energy up to 8.75 cm. This promotes nitric oxide release and thereby leads to vasodilatation of vessels, which stimulates the lymphatic system, increases circulation, removes toxins, and delivers higher levels of oxygen and nutrients to the injured cells [29]. Fostering the elimination of toxins and cellular waste products and helping to reduce inflammation. From another perspective, the infrared heat application keeps the episiotomy wound dry, absorbs fluids, plummets edema, prevents the growth of microorganisms, and hastens the cure of the episiotomy wound [22]. It also has the benefits of improving metabolism, helping in the regeneration of the body's cells, and developing pH in the body, so it can aid in healing damaged tissue [29].

The result of the current study is in agreement with the study [30] about the effectiveness of infrared lamp therapy in the healing of episiotomy wounds among postnatal mothers admitted to Adesh Hospital. They revealed that there was a significant statistical difference between the pre-test and post-test overall healing scores of episiotomy wounds between experimental and control groups. They concluded that the episiotomy wound healing is faster in the experimental group as the day progresses than in the control one. In addition, the present finding is consistent with a study conducted by Gomathi et al. [25] who revealed that infrared light application was effective in enhancing wound healing among postnatal mothers. Moreover, it is in accordance with a previously mentioned study by Rani [26]; who reported a significant decline in the mean wound healing score after infrared radiation therapy. Further, a similar result was observed in Khosla p (2017) research, which added that infrared radiation therapy is a simple and painless treatment when it is applied to the episiotomy injury site and painful areas because it promotes circulation, reduces inflammation, relaxes tissues, and enhances healing.

In contrast, Chandraleka et al. [19] concluded that using a sitz bath is more effective in improving wound healing among postnatal mothers than infrared ray therapy. In addition, Kalaivani [31] found that the application of the sitz bath is more effective in the experimental group based on REEDA parameters than in the control group. He concluded that the application of the sitz bath is effective in the episiotomy wound healing process. Surprisingly, Girsang and Elfira [32] revealed that the cold sitz bath hydrotherapy had a significantly greater effect in reducing perineal pain than the infrared heat application.

### 4.1. Limitations of the Study

The researchers faced the challenge of maintaining blindness during data collection where the raters were able to distinguish between parturient women in the study who received the dry and the control group who received warm water application. Although the current study showed a favorable impact of dry heat in promoting episiotomy wound healing and reduces their perineal pain, the study has limitations related to the small sample size. Further studies with larger sample sizes are needed to confirm the findings.

## 5. Conclusion

Application of the Primipara women of dry heat promotes episiotomy wound healing and reduces their perineal pain during early postpartum days than moist heat.

### 5.1. Recommendations

According to the present study's findings, it is advised that dry heat be included in the postnatal units' protocol as a useful nonpharmacological intervention to promote episiotomy wound healing and lessen perineal pain during the postpartum period.

## Figures and Tables

**Table 1:** Sociodemographic data of primipara women.

Characteristics	Dry heat group (*n* = 50)	Moist heat group (*n* = 50)	Significance
	No. (%)	No. (%)	
*Age (years)*			
18 < 20	6 (12.0)	10 (20.0)	*X * ^2^ = 3.278
20 ≤ 30	29 (58.0)	32 (64.0)	*P*=0.194
30–35	15 (30.0)	8 (16.0)	
Mean ± SD	26.44 ± 4.79	25.08 ± 5.01	*T* = 1.3916 *P*=0.167

*Education*			
Primary/preparatory	9 (18.0)	7 (14.0)	*X * ^2^ = 0.934
Secondary	30 (60.0)	28 (56.0)	*P*=0.627
University or more	11 (22.0)	15 (30.0)	

*Occupation*			
Housewife	46 (92.0)	43 (86.0)	*X * ^2^ = 0.919
Working	4 (8.0)	7 (14.0)	*P*=0.338

*Residence*			
Urban	9 (18.0)	14 (28.0)	*X * ^2^ = 1.412
Rural	41 (82.0)	36 (72.0)	*P*=0.235

*Type of family*			
Nuclear	27 (54.0)	30 (60.0)	*X * ^2^ = 0.367
Extended	23 (46.0)	20 (40.0)	*P*=0.545

*X* ^2^: chi-square test, *F*^ET^: Fisher's exact test, *T* (*P*): *T*-test, and *P* for *T*-test, significant at P ≤ 0.05.

**Table 2:** Total scores of perineal pain intensity before and after the intervention of dry and moist heat groups.

	Dry heat group (*n* = 50)			Sig	Moist heat group (*n* = 50)			Sig
	Before	5^th^ day	10^th^ day		Before	5^th^ day	10^th^ day	
	No. (%)	No. (%)	No. (%)		No. (%)	No. (%)	No. (%)	
*Total perineal pain intensity*								
No pain (0)	0 (0.0)	2 (2.0)	15 (30.0)	^MH^ ^*P*<0.001^ ^c^	0 (0.0)	0 (0.0)	0 (0.0)	^MH^ ^*P*=0.004^b^^
Mild (1–3)	6 (12.0)	12 (24.0)	20 (40.0)		7 (14.0)	3 (6.0)	30 (60.0)	
Moderate (4–6)	14 (28.0)	20 (40.0)	12 (24.0)		19 (38.0)	25 (50.0)	9 (18.0)	
Severe (7–9)	20 (40.0)	12 (24.0)	3 (6.0)		18 (36.0)	14 (28.0)	5 (10.0)	
Unbearable (10)	10 (20.0)	4 (8.0)	0 (0.0)		6 (12.0)	8 (16.0)	6 (12.0)	

^MH^ *P*: marginal homogeneity, test significant at ^b^*P* < 0.01^c^*P* < 0.001.

**Table 3:** Episiotomy wound healing assessment of dry and moist heat groups before and after the intervention.

Using REEDA scale	Before		Sig	On 5^th^ day		Sig.	On 10^th^ day		Sig
	Dry heat group (*n* = 50)	Moist heat group (*n* = 50)		Dry heat group (*n* = 50)	Moist heat group (*n* = 50)		Dry heat group (*n* = 50)	Moist heat group (*n* = 50)	
	No. (%)	No. (%)		No. (%)	No. (%)		No. (%)	No. (%)	
*Redness*									
None	0 (0.0)	0 (0.0)	*X * ^2^ = 0.622	2 (4.0)	0 (0.0)	*F * ^ET^	34 (68.0)	12 (24.0)	*F * ^ET^
Mild	21 (42.0)	24 (48.0)	*P*=0.891	36 (72.0)	27 (54.0)	*P* < 0.001^*c*^	16 (32.0)	30 (60.0)	*P* < 0.001^*c*^
Moderate	23 (46.0)	22 (44.0)		12 (24.0)	13 (26.0)		0 (0.0)	5 (10.0)	
Severe	6 (12.0)	4 (8.0)		0 (0.0)	10 (20.0)		0 (0.0)	3 (6.0)	

*Edema*									
None	0 (0.0)	0 (0.0)	*X * ^2^ = 2.36	27 (54.0)	11 (22.0)	*F * ^ET^	50 (100)	21 (42.0)	*F * ^ET^
Mild	16 (32.0)	18 (36.0)	*P*=0.967	16 (32.0)	25 (50.0)	*P* < 0.001^*b*^	0 (0.0)	23 (46.0)	*P* < 0.001^*c*^
Moderate	19 (38.0)	19 (38.0)		7 (14.0)	8 (16.0)		0 (0.0)	6 (12.0)	
Severe	15 (30.0)	13 (26.0)		0 (0.0)	6 (12.0)		0 (0.0)	0 (0.0)	

*Ecchymosis*									
None	11 (22.0)	8 (16.0)	*F * ^ET^	35 (70.0)	20 (40.0)	*F * ^ET^	47 (94.0)	30 (60.0)	*F * ^ET^
Mild	26 (52.0)	28 (56.0)	*P*=0.815	14 (28.0)	24 (48.0)	*P* < 0.007^*b*^	3 (6.0)	14 (28.0)	*P* < 0.001^*c*^
Moderate	10 (20.0)	12 (24.0)		1 (2.0)	5 (10.0)		0 (0.0)	6 (12.0)	
Severe	3 (6.0)	2 (4.0)		0 (0.0)	1 (2.0)		0 (0.0)	0 (0.0)	

*Discharge*									
None	50 (100)	50 (100)	*F * ^ET^	37 (74.0)	19 (38.0)	*F * ^ET^	46 (92.0)	29 (58.0)	*F * ^ET^
Serum	0 (0.0)	0 (0.0)	*P*=0.5	12 (24.0)	18 (36.0)	*P* < 0.003^*b*^	4 (8.0)	12 (24.0)	*P* < 0.005^*b*^
Serosanguinous	0 (0.0)	0 (0.0)		1 (2.0)	11 (22.0)		0 (0.0)	9 (18.0)	
Bloody/purulent	0 (0.0)	0 (0.0)		0 (0.0)	2 (4.0)		0 (0.0)	0 (0.0)	

*Approximation*									
Closed	50 (100)	50 (100)	*F * ^ET^	23 (46.0)	9 (18.0)	*F * ^ET^	47 (94.0)	30 (60.0)	*F * ^ET^
Mild	0 (0.0)	0 (0.0)	*P*=0.5	15 (30.0)	10 (20.0)	*P* < 0.001^*c*^	3(6.0)	9 (18.0)	*P* < 0.001^*c*^
Moderate	0 (0.0)	0 (0.0)		10 (20.0)	23 (46.0)		0 (0.0)	7 (14.0)	
Sever	0 (0.0)	0 (0.0)		2 (4.0)	8 (16.0)		0 (0.0)	4 (8.0)	

*X* ^2^: chi-square test, *F*^ET^: Fisher's exact test, significant at ^b^*P* < 0.01^c^*P* < 0.001.

**Table 4:** Total scores of perineal healing on the 5^th^ and 10^th^ days after intervention among dry and moist heat groups.

	Dry heat group (*n* = 50)		Sig	Moist heat group (*n* = 50)		Sig
	5^th^ day	10^th^ day		5^th^ day	10^th^ day	
	No. (%)	No. (%)		No. (%)	No. (%)	
*Total perineal healing score*						
Complete	39 (78.0)	48 (96.0)	^MH^ ^**P**<0.001^ ^**c**^	10 (20.0)	26 (52.0)	^MH^ ^**P**=0.031^ ^**a**^
Moderate	7 (14.0)	2 (4.0)		10 (20.0)	15 (30.0)	
Mild	4 (8.0)	0 (0.0)		16 (32.0)	8 (16.0)	
No healing	0 (0.0)	0 (0.0)		14 (28.0)	1 (2.0)	
Mean ± SD	2.34 ± 1.661	0.84 ± 0.739		6.24 ± 3.274	3.32 ± 2.645	
Sig	*Z * ^Wil^ = −3.46 (*P* ≤ 0.001^c^)			*Z * ^Wil^ = -2.15 (*P* ≤ 0.021^a^)		

^MH^ ^**P**^: marginal homogeneity test, *Z*^Wil^ = Wilcoxon signed ranks test, significant at ^a^*P* ≤ 0.05^c^*P* < 0.001.

## Data Availability

The authors confirm that the data supporting the findings of this research are available within the article.
